# ModuleOrganizer: detecting modules in families of transposable elements

**DOI:** 10.1186/1471-2105-11-474

**Published:** 2010-09-22

**Authors:** Sebastien Tempel, Christine Rousseau, Fariza Tahi, Jacques Nicolas

**Affiliations:** 1 IBISC, Tour Evry 2, 523, place des terrasses del'agora, 91000 Evry, France; 2 INP-ENSAT, Avenue de l'agrobiopole 31326 Castanet tolosan, France; 3 IRISA-INRIA, Campus de Beaulieu, bât 12, 35042 Rennes cedex, France

## Abstract

**Background:**

Most known eukaryotic genomes contain mobile copied elements called transposable elements. In some species, these elements account for the majority of the genome sequence. They have been subject to many mutations and other genomic events (copies, deletions, captures) during transposition. The identification of these transformations remains a difficult issue. The study of families of transposable elements is generally founded on a multiple alignment of their sequences, a critical step that is adapted to transposons containing mostly localized nucleotide mutations. Many transposons that have lost their protein-coding capacity have undergone more complex rearrangements, needing the development of more complex methods in order to characterize the architecture of sequence variations.

**Results:**

In this study, we introduce the concept of a *transposable element module*, a flexible motif present in at least two sequences of a family of transposable elements and built on a succession of maximal repeats. The paper proposes an assembly method working on a set of exact maximal repeats of a set of sequences to create such modules. It results in a graphical view of sequences segmented into modules, a representation that allows a flexible analysis of the transformations that have occurred between them. We have chosen as a demonstration data set in depth analysis of the transposable element Foldback in *Drosophila melanogaster*. Comparison with multiple alignment methods shows that our method is more sensitive for highly variable sequences. The study of this family and the two other families AtREP21 and SIDER2 reveals new copies of very different sizes and various combinations of modules which show the potential of our method.

**Conclusions:**

ModuleOrganizer is available on the Genouest bioinformatics center at http://moduleorganizer.genouest.org

## Background

A number of studies have described the search of repeated elements in a genome. However, except for phylogeny, few studies systematically analyze the relationships and variations between the copies of a given family of repeats.

TEs (Transposable elements) are present in nearly all genomes that have been studied to date and in some cases represent most of the genome [1]. These transposable elements move or are copied from one genomic location to another [2]. TEs are characterized and classified on the basis of terminal or subterminal remarkable structures or of their protein-coding capacity. TEs that encode the proteins involved in the amplification mechanism are called autonomous. Two types of amplification mechanisms define two classes of transposable elements. Class I elements, or retrotransposons, move via an RNA intermediate. Class II elements, or DNA transposons, seem to move via "cut-and-paste" mechanisms where the DNA element itself is the mobile intermediate [2].

The transposable elements have an important role in the evolution of eukaryotic genomes through their transposition mechanism [2,3] but also by their evolution/domestication [4-6]. Many recent studies clarify the diverse role of transposable elements in the evolution of their host genome: creation of NAIP protein isoforms and promoter by the insertion of L1 and Alu elements [3], plant light-sensing dependency on the presence of FHY1, FHL FHY3 and FAR1 that are related to MULE transposases [5], exaptation of the transposon CHARLIE10 in the mammalian zinc finger 452 gene [7] and creation of new host gene by capture of transposable element domains [4,6].

Many families of both classes do not show any coding capacity and are called non-autonomous transposable elements. They have cumulated so many mutations, insertions or deletions that these TEs are generally solely defined by their extremities [8,9]. Currently, most studies do not attempt to characterize and compare the internal sequences occurring between such extremities. A few methods [10-13] propose to segment sequences into conserved segments that we call modules, starting from a multiple alignment of these sequences.

**Figure 1:**
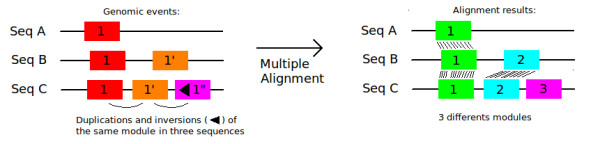
**Multiple alignment of duplicated modules**. Sequences A, B and C have duplicated blocks of nucleotides. These duplications evolved by mutation and the second duplication -1"- has reversed in the sequence C. Whatever the parameters, multiple alignment of the three sequences identifies the duplicated blocks as different modules.

Multiple alignments that find the boundaries of these segments in highly variable sequences like non-autonomous transposable elements may be hard to obtain. Moreover, multiple alignments lack to find duplication and inversion in sequences that are frequent in non-autonomous TEs (Figure 1).

In the present study, we propose a model and develop pattern matching and classification tools that allow identification, characterization and graphical representation of the combinations of modules that make up each sequence of a given family. We applied it to the study of a family of non-autonomous TEs of class II, called Foldback4 [14], in the whole genome of *Drosophila melanogaster*. This family has been chosen as an illustrative model of the complex internal organization of non-autonomous transposons, displaying a wide range of possible variations and a palindromic structure at the extremities of its sequences. We have also tested the method on other transposon families, namely AtREP21 (class II) [13] and SIDER2 (class I) [15], which confirm the interest of the tool we propose for the study of highly variable sequences.

## Methods

Our method represents a given family of TE sequences as an assembly of elementary blocks called *modules*. We propose an associated tool, ModuleOrganizer, assuming that these sequences have been selected on the basis of local characteristic features (for instance in a database such as Repbase [16]) and providing a global high level characterization of them facilitating the study of their variations. The section starts with a precise definition of properties that are suitable to delimit modules. We then describe in detail the method we propose for module identification.

Overall, it is based on the search and assembly of "*maximal repeat*" common to several sequences. A word *w *is a *maximal repeat *(*MR*) in a non-empty set of sequences *S *= {*S*_1_, ..., *S*_n_} if, and only if, there are *S_i_*, *S_j _*∈ *S *(not necessarily distinct) and letters *a*, *b*, *c*, *d*, with *a *≠ *b *and *c *≠ *d*, such that *awc *is a substring of $*S_j_*$ and *bwd *is a substring of $*S_j_*$ (where $ is a letter not occurring in any sequence). In order to compute all these MR, the sequences of the family are indexed via a generalized suffix tree [17-19]. Our algorithm recursively associates maximal repeats of a same sequence into modules under restrictions corresponding to their definition, such as their size, the number of sequences supporting their presence and the content of the sequence between two MR. Two final steps allow drawing an overall representation of the family: sequences are classified with respect to the presence or the absence of modules and a visualization tool yields an overall graphical view of the sequences.

### Defining modules in transposable elements

In theory all sequences of a given family of transposable elements are identical copies of an ancestor sequence. In practice an amount of variation is observed in TE copies, in connection with the age of the copies and the mutation rate. There are several kinds of TE that exhibit a reorganization of internal sequences including insertions and deletions of large sequences: the Miniature Inverted-repeat Transposable Elements (MITEs) [2,9], the Mu-related bacterial transposons [20,21] and the Helitron superfamily [22] that integrate blocks of genomic material into their variable sequence [21,23,24] and the Short Interspersed DEgenerated Retroposons 2 (SIDER2) [15].

Non-autonomous transposable elements (TEs that lost their protein-coding elements), like MITEs, which represent for some sequences the main source of copies, are often subject to deletions [25]. In such a case, it becomes difficult to reconstruct the autonomous element from the set of non-autonomous sequences [26]. We have studied as a test case the MITE family Foldback4 [14] and in accordance with previous studies of non-autonomous TEs [27,28], it clearly exhibits variations conserved across several sequences that could be largely explained by biological events such as insertions/deletions of mobile DNA or of host sequences [23,26]. In order to automatically retrace the main events that occurred, we have systematically exploited the fact that MITEs and other non-autonomous transposable elements present consensus patterns in their different copies [2,25]. For example, the MITE mPing, Foldback4 or AtREP21 share consensus extremities in all their copies simply because they are necessary for transposition [13,14,25]. The importance of host sequence acquisition mechanisms by TEs is well known in plants [29] and leads to detectable repeated blocks in copies separated by small non-consensus nucleotidic regions.

We propose a definition of module for this type of repeated blocks that introduces cautiously these separating nucleotides. Basically, a module is an assembly of flexible repeats. Each flexible repeat is a maximal repeat combination that occurs several times in sequences where MR are separated by a variable number of nucleotides. This class of repeats can be related to the class of *structured repeats *introduced by M.F. Sagot [30] but introduces new interesting variations that will be discussed in the **Results and discussion **section under paragraph **Structured versus flexible repeats**. Flexibility is founded on two simple criteria that delimit the possible spacers between consecutive repeats by fixing a reasonable level of similarity between instances of the same flexible repeat. Flexibility cannot be greater than the parts it links.

• *Flexible repeats*: Let *S *= {*S*_1_,..., *S_n_*} be a set of sequences. Let |*w*| denote the length of word *w *and *e*(*w*_1_, *w*_2_) denote the edit distance between words *w*_1 _and *w*_2_. A flexible repeat is inductively defined as follows:

1. Each maximal repeat is a flexible repeat

2. If *A *and *B *are flexible repeats and there exist a support subset of sequences *T *∈ *S *of cardinality at least 2, and words *A_i_x_i_B_i _*in each sequence *S_i _*of *T *satisfying the following constraints:

(a) *A_i _*and *B_i _*are occurrences of *A *and *B *in sequence *S_i_*

(b) Length condition: |*x_i_*| ≤ *max*(|*A_i_*|, |*B_i_*|)

(c) Distance condition: *e*(*x_i_*, *x_j_*) ≤ *min*(|*A_i_*|, |*A_j_*|, |*B_i_*|, |*B_j_*|) for all pairs *S_i_*, *S_j _*in *T*

then (*A*, *B*) is a flexible repeat with occurrences *A_i_x_i_B_i_*.

The definition recursively accepts chains of maximal repeats separated by variable constrained spacers. The length condition applies on spacers in each sequence individually whereas the distance condition requires a similarity level between all spacers globally. From this general notion of flexible repeat, one can define modules as a selection of flexible repeats that get a sufficient support in the set of sequences, that do not overlap and cover as much as possible of this set. More formally:

• *Modules*: Given parameters *MinSizeModule *and *MinSequences*, a module *M *in a set of sequences *S *= {*S*_1_, ..., *S_n_*} is a flexible repeat satisfying the following constraints:

1. Size condition: Each occurrence of *M *has length at least *MinSizeModule*.

2. Support condition: *M *is present in a support subset of cardinality at least *MinSequences *of *S*.

An *admissible set of modules M *= {*M*_1_, ..., *M_m_*} in a set of sequences *S *= {*S*_1_, ..., *S_n_*} is a set of modules such that:

1. Partition condition: For two different indices *i *and *j*, *M_i _*and *M_j _*do not overlap. Moreover, no other flexible repeat contains a module *M_i_*.

2. Maximality condition: No other flexible repeat fulfilling the previous three conditions (size, support and partition) could be added to *M*.

Such a definition aims at selecting globally a set of modules that must cover a largest subset of a set of sequences. Once admissibility has been reached, there remains some range of variation to build a set of modules from a set of sequences. We propose an iterative strategy based on a preliminary search for seeds at the core of the largest flexible repeats.

### An assembly algorithm for the creation of modules

Targeted modules have sizes greater than *MinSizeModule *and are present in at least *MinSequences *sequences. All admissible modules are based on an assembly of maximal repeats. In an initial step, our algorithm will thus build the set of all MRs present in at least *MinSequences *sequences. This may be achieved in linear time with respect to the cumulated length of the sequences, using a generalized suffix tree [19]. These exact maximal repeats can be considered as seeds which are extended to the left or to the right depending on the admissibility of the extension. This method of seed extension is similar to the method used in Blast [31].

The construction of modules is detailed in Algorithm 1. Its basic data structure is a list *L *of MR sorted by decreasing size, then by number of occurrences. Each maximal repeat is associated with the sorted list of its occurrences in increasing position. Initially, *L *contains the whole set of MRs present in at least *MinSequences *sequences and it is updated after the construction of each module (line 8 and 11 in Algorithm 1).

#### Algorithm 1

1. BuildModules(L, *MinSequences*, *MinSizeModule*)

2. REQUIRE: Sorted list L of possible MR (size m, decreasing order)

3. REQUIRE: Minimal Number of covered sequences *MinSequences*

4. *i *← 1; *PairOk *← FALSE

5. *COMMENT*: *Looking for a a pair of MR (Seed, Next) in decreasing order of size in L*

6. WHILE (*i *<*m *and not *PairOk*)

7.   *Seed *← *L*[*i*]

8.   (*Next*, *PairOk*) ← BuildPair(*L*, *Seed*, i+1, MinSeq)

9.   *i *← *i *+ 1

10. IF (*PairOk*)

11.   Discard the paired occurrences *A *of *Seed *from *L*

12.   *COMMENT*: *Try to enlarge the current flexible repeat to the left or to the right by a new maximal repeat*

13.   WHILE (*PairOk*)

14.      Depending on the observed occurrences of the flexible repeat, replace *Seed *by *SeedXNext *or *NextXSeed*

15.      Discard the paired occurrences *B *of *Next *from *L*

16.      *PairOk *← FALSE

17.      (*Next*, *PairOk*) ← BuildPair(L, Seed, 1, MinSeq)

18.   IF (size(*Seed*) ≥ *MinSizeModule*)

19.      Seed and its occurences as a new module

20.   ELSE ∅

For each module, the algorithm considers the largest remaining MRs as seed candidates and looks iteratively and greedily at flexible repeats that can be built from such seeds. At line 8 of Algorithm 1, a flexible repeat made of a flexible repeat *Seed *and a maximal repeat *Next *has been discovered and will serve as a new seed for the search of larger flexible repeats (line 10). Once it is not possible to extend it any more (line 14), the last condition to be checked is the size of the obtained module.

**Figure 2:**
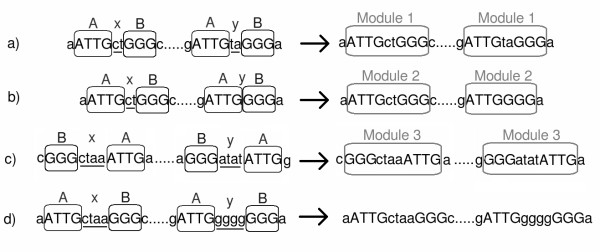
**Examples of association of maximal repeats (MR)**. The MRs *A *and *B *are surrounded by rectangles. The spacer sequence between two MRs is denoted *x *or *y *and is underlined. Modules, created by the assembly of *A*, *B *and *x *or *y*, are surrounded by large rectangles. Case a) is the most simple case of association of maximal repeats *A *and *B*: the distance between them is always smaller than the size of the smallest MR *B *and they can be safely associated. Case b) shows that two occurrences of MR can be separated by any sequence, including the empty one. In case c), the distance between occurrences of *A *and *B *is equal to the size of the largest maximal repeat and spacers have to be checked for their similarity. The edit distance between sequences *x *and *y *equals 2, a value smaller than the sizes of A and B, and a module can be built. In case d), the size of *x *and *y *equals 4 as in case c), but *x *is at edit distance 4 from *y*, which is greater than 3, the size of *B*. The association of MRs *A *and *B *do not form a module in this case.

The search for maximal repeats to be associated with seeds in flexible repeats is described in Algorithm 2. Associations are represented as *AxB *or *BxA*, where *A *is the largest part and *B *is the smallest part of flexible repeats. This convention explains the simplified tests for flexible repeat length and distance in Algorithm 2. The spacer *x *can be an empty sequence (Figure 2). The first condition of flexible repeats is checked in line 5 and the second in line 11 (Algorithm 2). The test in line 11 also checks if there is at least one association in each of *MinSequences *sequences, the first condition for a flexible repeat to be retained as a potential module. In building flexible repeats, the algorithm chooses the largest maximal repeat *B *that has the most associations with *A*.

#### Algorithm 2

1. REQUIRE: BuildPair(L, Seed, Start, *MinSequences*)

2. REQUIRE: Sorted list *L *of possible MR (size *m*)

3. REQUIRE: A flexible repeat *Seed *and a starting index *Start *for the search in *L*

4. REQUIRE: Minimal Number of covered sequences *MinSequences*

5. *j *← *Start*; *PairOk *← FALSE

6. WHILE (j≤m and not *PairOk*)

7. *Next *← *L*[*j*]; *Pairs *← Ø

8.   FOR (all occurrences *A *of *Seed*)

9.      Search for the occurrences *B *of *Next *such that *AxB *(orientation = "+") or *BxA *(orientation = "-") is a subword of the sequence and x has size at most the size of *A*

10.      IF (*B *exists)

11.         *Pairs *← *Pairs *⋃ {(*x, b, orientation*)}   *COMMENT: b is the size of B*

12.   *COMMENT: Build the graph GB of occurrences at a suitable edit distance in Pairs*

13.   FOR (all pairs of occurrences ((*x*, *b_x_*), (*y*, *b_y_*)) in *Pairs *in each orientation

14.      IF (the edit distance *e*(*x*, *y*) ≤ *min*(*b_x_*, *b_y_*))

15.         Create an edge in *GB *between vertex (*x*, *b_x_*) and vertex (*y*, *b_y_*)

16.   IF (there exists a clique containing at least *MinSequences *sequences in *G*)

17.      *PairOk *← TRUE

18.   *j *← *j *+ 1

19.   RETURN (*Next*, *PairOk*)

The worst case complexity of Algorithm 2 is o(*n*^3^), where *n *is the cumulated size of sequences: it is based on a loop on possible maximal repeats (o(*n*)) including a loop on possible matching occurrences (o(*n*) since list of occurrences of *A *and *B *can be searched in parallel), the production of a graph of similar occurrences (o(*n*^2^)) and a search of a clique of size at least *MinSequences *in this graph (we use a heuristic search here, keeping only vertices that are connected to at least *MinSequences *nodes associated to different sequences and using an iterative choice of vertices with highest output degree in the remaining graph. The complexity of this step is thus o(*n*)). Note that the last step offers no guarantee to always find the clique if it exists. Algorithm 1 is also based on a loop on possible maximal repeats (o(*n*)) including calls to Algorithm 2. The total worst case complexity is thus o(*n*^4^). The main data structure are the list *L *of occurrences of maximal repeats and the graph *G*, requiring o(*n*^2^) space. In practice, the algorithm is very fast (less than a minute) on typical transposable elements families (e.g. 15 sequences of length 2000 nt). The tool we propose allows in fact a more flexible method of association of *A *and *B*. The size of spacer *x *has to be smaller than a percentage of the size of *A *(|*x*| ≤ |*A*| * percentage). By default, the percentage value is 100% and corresponds to the criterion we have defined. With a lower percentage, it is possible to be more restrictive on the spacer size.

### Detection of all modules in sequences

After the creation of a module, the list *L *of maximal repeats must be pruned of any occurrence that overlaps occurrences of this module in order to fulfill condition 3 of the definition of modules (partition condition). The algorithm stops the search for modules present in *MinSequences *sequences when the procedure BuildModules returns an empty set.

At the beginning *MinSequences *is set to the number of sequences present in the input file. After the algorithm has found all modules of size bigger than *MinSizeModule *in *MinSequences *sequences, the main loop searches for modules in *MinSequences *= *MinSequences - *1 sequences until *MinSequences *= 1.

### Palindromic modules and truncated modules

Transposable elements have two characteristics that are not taken into account by our module definition. First, elements may be copied in the direct or reverse direction. This leads to frequent palindromic motifs that have to be recovered in the context of flexible repeats. Second, elements may be truncated due to large deletions or a high number of mutations. Some partial flexible motifs may remain interesting to identify for a complete analysis of the transposon structure.

Our tool proposes the identification of reverse modules on the basis of the exact MR they contain. In practice, all MRs are searched both on the direct and on the reverse strand and are consequently labeled. A reverse module has the same composition as the module it is derived from, replacing its MR by the corresponding reverse MR. Once a module has been determined, the presence of its reverse module is systematically looked for in the sequences. The presence of a module in its direct or reverse form is counted whatever its direction. This way, the requested number of supporting sequences *MinSequences *may be attained by a combination of both directions.

Truncated modules may exist with a conservation level that is very low, one or several MRs being discarded from the original copy. It is difficult to define an absolute conservation threshold that would decide if a given degenerated combination of MR remains or not a truncated version of a module or if it must be considered as a new entity. Modules are often composed in practice by a main founding MR surrounded by several smaller MRs at some distance. We have chosen to require that the largest MR remains present in a truncated version of a module, a simple constraint that ensures at least a core identity with the full module. Unlike the previous case, truncated modules are considered only if the full module exists in the set of sequences. They are added during a second step, once all complete modules have been identified.

### Clustering of sequences

After the module detection stage, assume *m *different modules have been obtained in *n *sequences. The next step of ModuleOrganizer is to build a hierarchical clustering of sequences (a tree) conducted on the basis of module similarity. This way, the evolution of sequences in the family can be traced back by comparing sequences in decreasing order of similarity of their modular profiles. Basically, each sequence is represented by a vector of values on a set of attributes (variables), one numerical attribute per module that is a counter of its occurrences. If the user does not select the search for reverse or truncated modules, the software creates thus an incidence matrix of dimensions *m *× *n*.

If reverse modules are allowed, more attributes are necessary to finely describe the evolution of sequences. For each module we create three attributes: one counting the number of direct occurrences (direct orientation), one counting the reverse occurrences, and a last counter for occurrences of the module (either normal or reverse occurrence). The third attribute allows to measure the convergent evolution of palindromic structures like Inverted Terminal Repeats [32]. Indeed, the transposase of autonomous elements recognizes specific palindromic structure at their extremities [2]. The mutation of one extremity decreases strongly their transposition. A double mutation in both extremities may restore the palindromic structure and transposition. This has been observed for instance within families of mariner-like elements [33]. The incidence matrix has size 3*m *× *n *with the reverse option.

**Figure 3:**
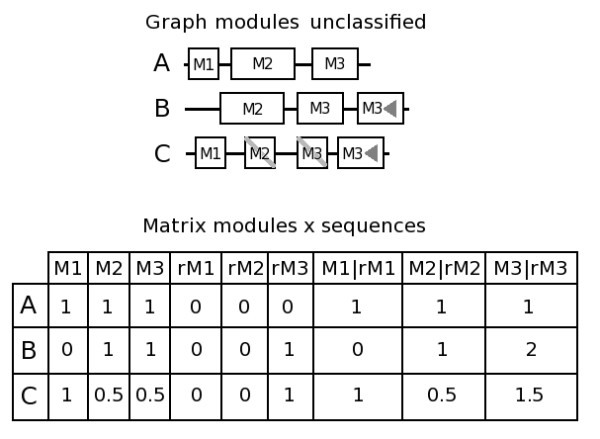
**Module organization of sequences A, B and C and associated incidence matrix used for the classification**. Modules marked with triangles correspond to reverse modules and crossed modules correspond to truncated modules. Three attributes are associated with each module, *Mi*, *rMi *and *Mi*|*rMi*, corresponding to direct, reverse and total occurrences. Truncated modules have 50% of the MR total size conserved.

The presence of truncated occurrences of modules slightly extends the meaning of the attributes we have just defined. These occurrences correspond to small fragments of entire modules, and are composed of a selection of the module MR. While full module occurrences contain 100% of the cumulated size of MR, truncated occurrences contain a lower percentage of this total size reflecting the deleted fraction of a module MR: a complete direct or reverse occurrence will contribute for 1 and a truncated occurrence will have a strictly smaller positive contribution. For instance in Figure 3, the module M2 has one complete occurrence in sequence A and B and one truncated occurrence that contributes at level 0.5.

Sequences are clustered using a standard Hierarchical Agglomerative Clustering (HAC) algorithm, using the Ward criterion [34]. Ward's criterion states that merging HAC clusters should be focused on minimizing the increase of variance induced by the added interclass variance. Basically, it is an error sum-of-squares criterion. In the first step, the loss of inertia in aggregating sequence pair *x *and *y *$\Delta (x,\;y)=\frac{1}{2n}{\sum_{i\in attributes}\left(x_{i}-y_{i}\right)}^{2}$ is computed between each possible pair, where *x_i _*(*y_i_*) corresponds to the value of attribute *i *in sequence *x *(*y*) and *n *is the number of sequences. Starting from clusters reduced to a single sequence with weight 1/*n*, the pair of clusters minimizing Δ is replaced by its union and the values of the weight and Δ for this new cluster is updated (weights are additive and if *x*, *y *and *z *are three clusters with weight *n_x_*, *n_y _*and *n_z_*, then $\Delta (x\cup y,\;z)=\frac{1}{n_{x}+n_{y}+n_{z}}((n_{x}+n_{z})\Delta (x,\;z)+(n_{y}+n_{z})\Delta (y,\;z)-n_{z}\Delta (x,\;y)))$. The algorithm iterates until all sequences are in the same cluster.

## Results and Discussion

### Implementation

**Figure 4:**
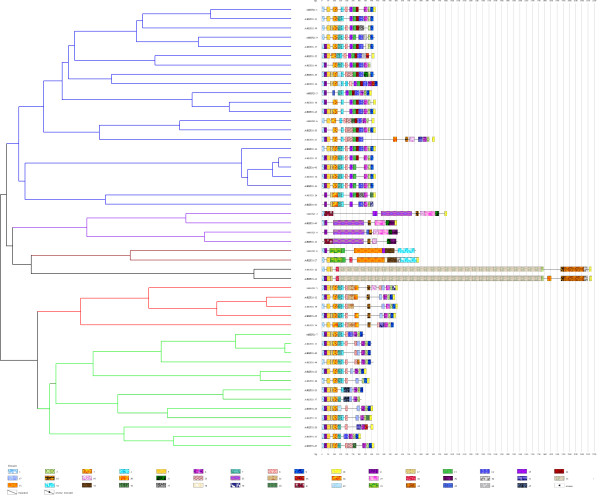
**Module organization of the AtREP21 family**. The 48 sequences of AtREP21 are composed of 47 modules. The only parameter used is *MinSizeModule *= 18. The sequences are classified according to their module composition and the tree appears as displayed by ModuleOrganizer. We just added colors to the hierarchical tree in order to highlight the clusters.

The module detection program (all its functions) and the classification program are written in the C language. The software produces one to three output files. The first file is the only mandatory output file. It corresponds to the list of sequences with their composition in modules. It uses the same output format as DomainOrganizer [13]. The second file corresponds to the classification of sequences and is written in Newick format. The third file is the output graph written in SVG (Scalar Vector Graphics) format (Figure 4). In this file, the sequence module contents are displayed together with their classification tree. A specific texture is associated to each domain and specific markers are used for reverse (triangle) or truncated (crossed boxes) modules.

ModuleOrganizer is relatively fast. The next two sections give results on three transposable element families: AtREP21, SIDER2 and Foldback4, for a total of 27000, 8000 and 30000 nucleotides respectively. On a PC running with an Intel Core2 Duo 2.2 GHz and 4 Go Ram, these results have been obtained in 15.7 s, 1.8 s and 27.7 s respectively.

ModuleOrganizer allows users to tune a few parameters with respect to their application:

• The first parameter is the minimal size *MinSizeModule *of admissible modules. We assume that in most cases the user has some knowledge of the sequences in the input file so that the user can fix or adjust iteratively the value of *MinSizeModule*. By default, ModuleOrganizer proposes to set *MinSizeModule *to the minimal value of *x *such that it exists a word of size *x *that is not present in the sequences.

• The second parameter *Maxratio *is the percentage that can be used in the length condition of flexible repeats (the length condition of flexible repeats writes |*x_i_*| ≤ |*A_i_*| ∗ *Maxratio *if *A_i _*is assumed to be larger than *B_i_*), e.g. *Maxratio *= 100 corresponds to spacers between elements *A *and *B *of flexible repeats possibly reaching the size of *A*. The selection of modules is a trade-off between size and similarity. Lower values of *Maxratio *allows to move this trade-off to smaller sizes of modules and more similar occurrences (see end of section "An assembly algorithm for the creation of modules").

• The third parameter is the minimal number of sequences that must support the presence of modules. Our algorithm searches modules in *MinSequences *sequences. By default, *MinSequences *will get all values in the range from the number of sequences up to 1 sequence.

• The last parameter allows the search for palindromic and/or truncated modules (see section "Palindromic modules and truncated modules").

### Module organization of AtREP21 and SIDER2

AtREP21 is a family of non-autonomous Helitron transposable elements present in *Arabidopsis thaliana *[13]. We used ModuleOrganizer on the 48 elements of this family, setting just the *MinSizeModule *parameter to 18 instead of the default value 8 that would result in a too detailed view. No palindromic modules exist in this family [13] and it is not necessary to use this optional search. There are 47 modules that characterize the whole family (Figure 4). The module number 1 and 10 correspond respectively to the left and right extremities of AtREP21. The comparison of results between DomainOrganizer [13] and ModuleOrganizer shows a similar module organization. For example, the AtREP21 elements number 5, 12, 19, 25, 34 belong to the same cluster (red group in [13] and Figure 4). The sequences of AtREP21-24 and AtREP21-41 are clustered by ModuleOrganizer in a specific group that corresponds to the insertion of a long transposable element. This is the sole minor difference between results provided by the two methods. For this family, the main difference lies rather in the execution time: more than 4 hours for DomainOrganizer and less than one minute for ModuleOrganizer.

**Figure 5:**
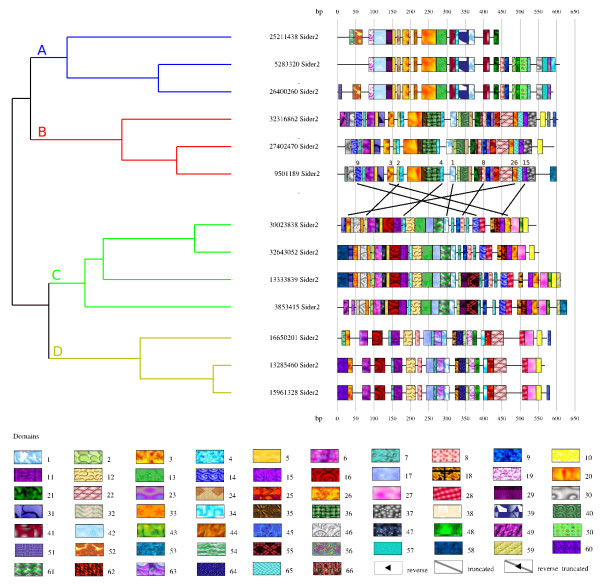
**Module organization of the SIDER2 family**. The 13 sequences of SIDER2 are composed of 58 modules. The parameters used are *MinSizeModule *= 10 and *Maxratio *= 75. The value of this last parameter allows to create smaller and more similar modules than by default. We added colors and letters to the hierarchical tree produced by ModuleOrganizer that highlight the clusters. We also switched the clusters to show the crossing modules.

SIDER2 is a family of Short Interspersed DEgenerated Retroposons that Smith *et al*. found in three *Leishmania *genomes [15]. We selected 13 of the 1021 SIDER2 they reported. We set the parameter *MinSizeModule *to its default value 8 and the parameter *Maxratio *to 75, a value that generates a more detailed segmentation with more similar module occurrences than the default value 100. This allowed to better recover crossing effects that we wanted to show. The Figure 5 shows SIDER2 sequences are clustered into four groups: A, B, C and D (independently of the value of *Maxratio*). Group A is mainly composed by the modules 19, 42, 29, 26, 20, 13, 39, 1 and 41 (5'-3' order). The larger modules of SIDER2 sequences of group C are modules (5'-3' order) 26, 46, 6, 16, 29, 4, 17, 61, 1, 8, 14, 28, 18, 62, 15 and 27. In the SIDER2 sequences of group D, modules 29, 55 have been deleted and modules 13 and 61 have been substitued by 8 and 63 respectively. Group B presents a clear combination/exchange of modules with the other groups: the modules 9, 6, 25 and 22 come from the C/D groups and the module 20 from group A. Moreover, Figure 5 shows some conserved modules that would be impossible to observe with multiple alignment: some modules cross themselves in SIDER2 sequences. For example, module 1 is present in all sequences: in group B it lies between modules 9 and 3 and the module 26, but in group C it lies between module 26 and the modules 9 and 3.

### Organization of modules in Foldback4

We have chosen the family of non-autonomous TEs Foldback4 (FB4) known to be present in the *D*. *melanogaster *genome [14] in order to propose a more complete comparative analysis of a TE family with several available softwares.

**Figure 6:**
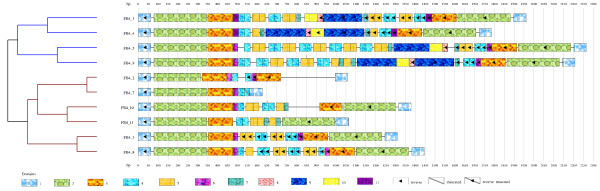
**Module organization of the Foldback4 family**. The ten sequences of Foldback4 are composed of eleven modules. To obtain this result, *MinSizeModule *has been set to 18 and truncated, and reverse modules have been looked for. We added colors to the hierarchical tree produced by ModuleOrganizer that highlight the clusters.

Ten Foldback4 sequences have been identified and numbered with respect to their order of occurrence on the direct strand. These elements range from 627 bp to 2266 bp. We used STAN (Suffix Tree Analyser) [35] to find the sequences from the genome and FGENESH software [36] to verify that sequences do not contain ORFs and are thus non autonomous elements. *MinSizeModule *was set to 18 bp and we have used the optional search for palindromes and truncated modules in sequences. The module-identification algorithm discovered 11 modules (Figure 6).

The palindromic structures of the Foldback4 family have been recovered and are described by domain numbers 1-3 and 6. Except for Foldback sequences number 2 and 7, which present a deletion of modules 2, and 2-3 respectively, termini palindromes of this family measure more than 400 bp and represent the most important part of their sequences (Figure 6). The Foldback4 family shows a significant variation in internal sequences resulting from insertions, deletions or substitutions of domains. The visualization shows that Foldback4 is mainly divided into two groups of sequences.

The first group (color blue in Figure 6) contains Foldback4 sequences number 1, 4, 5 and 9. This group is mainly composed of the combination of modules number 1-5, 7, 9-10 and their reverse complement. The most notable variation between them comes from the number of repetitions of modules 4 and 5 that represent the central part of these sequences. The sequence FB4_4 does not contain any repetition and FB4_9 contains three consecutive repetitions of modules 4 and 5.

The second group (color red) contains Foldback4 sequences number 2-3, 7-8, 10-11. It is mainly characterized by the deletion of modules: no sequence of this group does contain modules 8-10 in its middle part. The module 2 that belongs to the left side of the palindromic structure is deleted in sequences 7 and 2. The sequence 7 contains only the module 1 in the right part of the palindrome and the module 2 is deleted in the right part of the palindrome of sequence 2.

Recovering the module architecture in the Foldback4 sequences establishes a probable scenario for the evolution of this family. Note that we do not propose here a complete phylogenetic analysis of the family that would be beyond the scope of this paper. Our purpose is just to illustrate the kind of hypotheses that can be elaborated from such characterizations. Like MITE elements, non-autonomous families generally derive from autonomous elements that are subject to deletion events [9]. After such deletions, the amplification of TE can create tandem repeats of minisatellites inserted into the non-coding sequence [2]. For the Foldback4 family, we assume similar deletions and mutations have created these non-autonomous elements from autonomous elements and that in a second step some internal duplications have occurred. If it is the case, the oldest sequence (the closest from the autonomous) contains the highest number of modules together with the lowest number of duplications. The sequence FB4_4 corresponds to these criteria: it contains both all modules and the least number of repeated modules. We then assume the evolution of this family mainly comes from the duplication or the deletion of modules. From this sequence FB4_4 other sequences have evolved by the amplification of left internal modules 4 and 5 (Figure 6). The sequences 5 and 9 seem older than the other sequences because they did not evolve after these amplifications. From these sequences, the amplification of the right modules led to the sequence 1. The other sequences then evolved from this point by the deletion of the central modules. We assume that sequences 3 and 8 have been created from sequence 1 by the deletion of one block of consecutive modules (just one deletion event).

Our method highlights the complexity and variance of the sequence structure between members of a given family of TEs. These differences, which may result in a certain level of disconnection between TE repetition and module repetition within a genome, are usually not observable by standard sequence analysis tools on DNA such as BLAST, or specialized software programs for the analysis of TEs (Recon [37], RepeatScout [12], Vista [10] or GATA [11]).

### Structured versus flexible repeats

The idea of looking for a series of words placed at constrained distances on genomic sequences is not new. The class of *structured repeats *has been introduced in [30] in order to formalize the type of sequences involved in regulation (binding sites). Basically, a structured repeat is define as an ordered collection of several simple motifs (*the boxes*) interleaved by bounded gaps.

*Spaced dyads *are probably the simplest notion of structured motif and have been proposed by J. van Helden & al [38]. A spaced dyad is made of two boxes, two words at a fixed distance. The more general notion of structured motif as it is used for instance in Risotto [39] allows variations in the size of single motifs (specified minimal and maximal length for each), in the content of single motifs (specified maximal error rate for each) and variation in the size of gaps (specified minimal and maximal distance between each pair of consecutive box). The number of motifs is fixed and the search for structured motifs uses a quorum corresponding to our parameter MinSequences.

Numerous authors have applied structured motifs in the framework of pattern matching (motifs are given) and this differs significantly from the pattern discovery task we are considering in this paper where the combinatory of possible arrangements has to be carefully controlled. The language A of ANREP [40] is one of the early successful proposition in this respect, already distinguishing simple motifs (including the possibility to manage matching costs) from *network patterns *that allow gaps and disjunctions. Advanced algorithms have been designed since and a few papers have presented experiments on the analysis of transposable elements [41-43].

Flexible repeats introduce a few specific characteristics with respect to structured repeats. First flexible repeats are not vectors of fixed size: flexible repeats may have a variable number of boxes and they are included in an embedding structure that is not a simple linear ordering. Second, the possible variations are strictly confined in the gap part (*x *part in flexible repeats). Boxes are words (maximal repeats) that have to match exactly in the sequence. The restriction to MR provides a representative basis of all words that has a linear size with respect to the cumulated length of the sequences. Note that there is no restriction on the length of these words and in practice all short words including single nucleotides are maximal repeats. Structured repeats present a two level view of sets of sequences, a level of similar regions shared by a sufficient subset of these sequences and a level of assembly with completely specific regions that are solely represented by their length. We have adopted a different view where only the regions *exactly *shared by a sufficient subset of sequences are retained and where the assembly process results from a cautious iterative aggregation of these regions on the basis of similar inter-regions. Regions that are completely specific to particular sequences are not associated with a module and are thus characterized negatively by the absence of modules.

To sum up, the standard approach for the segmentation of biological sequences in relevant modules is the pattern matching approach, where one is looking for common words within a certain admissible rate of errors. The issue described in this paper in rather an aggregation approach: starting from solid anchor boxes (the MR), we establish reasonable criteria to put them in a same class, in a user-understandable way. This is why we use the length of these anchors in our criteria. Dropping the first condition on length in the definition of flexible repeats results in uncontrolled aggregations: it leads to a single module spanning the whole sequences for the family Foldback4 for instance. Looking for *AxB *with the number of allowed errors made proportional to |*x*| in the second condition instead of *min*(|*A*|, |*B*|) cannot be retained because among possible *A *and *B *are single nucleotide sequences and this would results in simply looking for all common approximate words on the set of sequences, a hard problem that has not been answered satisfactorily so far, as it appears in the following discussion comparing results of available tools. To the contrary, the principle "flexibility cannot be greater than the parts it links" offers a neat basis of aggregation that can be practically finely tuned by using parameter *Maxratio*.

### Comparison with other programs

Studies on non-autonomous transposable elements are rare because the main method of studying the evolution of these elements is based on their alignment with other known elements. Since non-autonomous elements are generally highly variable, including numerous insertions, deletions, and repetitions, previously cited methods fail to find a good organization of the non-autonomous elements. We have tested four recent softwares that combine multiple alignment and graphical tools on the Foldback4 family, trying each time to retrieve direct or reverse copied modules: VISTA [10], GATA [11], GraphDNA [44], Recon [37] and DomainOrganizer [13]. Graphical results of these software are provided in the additional files.

The first software, VISTA http://genome.lbl.gov/vista/index.shtml[10], requires entering sequences separately and choosing a type of alignment. The user cannot set the value of multiple alignment parameters and one of the sequence must be set as a reference. In our case, the user has to check *n *results where *n *is the number of sequences. Moreover, as VISTA does not display the reference sequence, it is impossible to obtain a complete view of all sequences. We provide in Additional file 1 the graph resulting from the selection of F4_1 as the reference sequence. The graph shows that, except for FB4_7, both extremities are conserved in all Foldback4 sequences and that some parts of the internal sequence are conserved. We obtained similar results with the other FB4 sequences as reference. VISTA does not provide the palindromic structure of Foldback that is important to understand the evolution of these sequences. Moreover, the VISTA interface does not allow to change the parameters of the matching region (the minimum size is 100 bp and the minimum percentage of similarity is 70%) and the complexity and the structures of Foldback4 internal sequences vanish completely.

GATA http://gata.sourceforge.net[11] contains two different softwares: GATAliner and GATAPlotter [11]. GATAliner uses Blast2seq [45] to create the alignment. Beside standard parameters of BLAST, such as the seed size and the mismatch cost, it offers a specific parameter on the minimal size of alignments that corresponds to *MinSizeModule *in our algorithm. GATAPlotter is an interactive graphical tool that shows the alignment of a reference sequence (like VISTA) against another sequence. GATAPlotter has lots of rendering options and provides also two interesting options for the analysis of the dataset: annotation of aligned sequences and matching regions displayed by percentage of similarity. Additional file 2 assembles all multiple alignments with a fixed threshold of 50% similarity to the reference sequence. The first sequence of each alignment corresponds to the reference sequence. On the contrary of VISTA but like our algorithm, GATA shows these sequences contain a large palindromic structure at the extremities. Except the FB4_4 and FB4_9, all other sequences have a mismatch region in the center of their sequences. The main limitation of GATA is the number of sequences the user can study. The user must open *n*^2 ^windows for *n *sequences to get a global view of them. For Foldback4 sequences we needed to open 100 different alignments. In practice, studies with more than 5 sequences become tedious.

GraphDNA http://athena.bioc.uvic.ca/tools/GraphDNA[44] uses fixed parameters for the multiple alignment. GraphDNA provides many views of the alignment such as 'Purine Skew', 'AT Skew' and 'DNA Walker'. These views show the skew of nucleotide combination at a given position, calculated on a window of user-defined size. DNA Walker is a graphical view of the four nucleotide skews along the dataset sequence. We chose the 'Purine Skew' view for the analysis of our dataset (Additional file 3). GraphDNA provides in this case a global view of similar nucleotide composition regions that fit well with the conserved blocks observed by using a multiple alignment procedure. Contrary to the two previous softwares, no reference sequence is needed and all sequences are displayed within a single view. We assumed that similar or parallel curves denote similar sequence fragments. The graph shows that all left extremities of sequences, except FB4_1 and FB4_2, start at the same point, and are similar (Additional file 3). After about 200 common bp, the sequences start to take different skews (directions), and after 300 bp, sequences completely diverge. The right extremities are parallel and present the same skew pattern. This suggests that right extremities are similar too but give no details on the internal part.

Recon http://selab.janelia.org/recon.html[37] is a Perl script using BLAST results [31] as input. The software aggregates the different fragments of BLAST hits in one long aligned region. Users cannot tune the parameters of Recon. On our dataset, the software chose Foldback4 number 11 as the reference sequence.

Two results of Recon are presented: the final result with all similar parts assembled in blocks after the complete execution of Recon algorithm, and an intermediate state given by Recon that corresponds to the similar parts identified by BLAST and sorted by Recon. The Additional file 4 displays the final result using ModuleOrganizer textures. It shows 11 modules that often cover a large part of the sequence. These large modules do not show palindromic structures and differ for each sequence. However, the module number 2 is overlapped by modules 3 and 8 in FB4_1 and FB4_5 respectively. Because we needed details on the composition of each sequence, we had to use the intermediate results proposed by Recon, which contained the unclustered fragments. First, we removed the fragments shorter than *MinSizeModule *(i.e. 18 bp), we labeled the fragments of each Foldback TE in agreement with fragments in the reference sequence and we sorted the fragment by increasing positions. Finally, we renamed modules having the same coordinates in Foldback sequences but not in the reference sequences and we also associate manually modules with different names that have similar coordinates in the reference sequence. The Additional file 5 shows the fragments detected by Recon. The Recon modules (Rmodules) 1, 30, 117, 165, 174 and 182 show the relative similarity of left extremities. Right extremities are similar too and share common Rmodules with left extremities, suggesting they could be complementary sequences. It is extremely difficult to point at similarities or structures in the internal part of sequences with the overlapping fragments, especially in the FoldBack4 sequence 11 that contains all Rmodules. For example, only FB4_5 exibits some repeated Rmodules in its internal sequence.

DomainOrganizer [13] fails to give a result with the Foldback4 sequences. During the crucial and costly step of domain optimization, too many candidate domains are generated and the program is unable to find out a possible cover of sequences with domains (unpublished data).

GATA [11] is the only tested software that showed the palindromic structure. All other softwares failed to show the real structure of extremities and the module evolution of Folback4 sequences. Especially the internal part of sequences remained 'black boxes' in all these tools. Except for DomainOrganizer where we were forced to use the AtREP21 family for the comparison, we summarize in Table 1 the range of application of each software extracted from our study on Foldback4.

**Table 1:** Summary structures detected with softwares

	Duplication	Palindrome	Truncated	Reference Sequence
ModuleOrganizer	X	X	X	

DomainOrganizer	X			

VISTA				X

GATA		X		X

GraphDNA				

Recon	x	x		X

The table summarizes the different structural modules that can be detected by the different tested softwares. The last column indicates if the software needs a reference sequence or not. Big crosses in cells indicate the software displays the corresponding type of structure, small ones mean the structure could be deduced from the results.

## Conclusions

Our analysis provides structural results on the internal organization of a family of DNA sequences. It can describe the differences between family members in terms of module content and highlights the evolution of the host genome with respect to these components. Such a structural and descriptive abstract view should ease the analysis of TE-genome relationships and give some support for studies on transposition mechanisms.

Our method needs very few parameters. The most crucial one is *MinSizeModule *and tuning this parameter with several tries is generally sufficient to get desired results. If the size of minimal domains is too small, the number of domains may simply be too large to give an interesting abstraction of the sequence. On the other hand, if the size of domains is too large, the number of domains may be too restricted to formulate a relevant biological interpretation.

Of course, ModuleOrganizer might be applied in principle to any set of nucleic sequences sharing some similarities and for which a multiple alignment fails to correctly retrieve the architecture of conserved blocks in the sequences. The study of families of transposons is a natural setting for this tool but application on other types of sequences might help to explore other sequences at the desired level of abstraction.

## Authors' contributions

ST has produced all the necessary code with the help of CR and made the experiments. JN supervised the study and proposed the model. JN and ST wrote the initial version of the paper. CR and FT have checked and improved the writing of the paper. All authors read and approved the final manuscript.

## Supplementary Material

Additional file 1 **Visualization of Foldback4 family with VISTA**. We chose FB4_1 as reference sequence. The bright red zones correspond to high similarity regions and the white zones correspond to low similarity region. The alignment criteria of VISTA are fixed in the software. Click here for file

Additional file 2 **Visualization of Foldback4 family with GATA**. The reference sequence is FB4_1. The black and gray rectangles correspond to regions matched in the same direction and the red and bright red ones correspond to regions matched in reverse direction. The brighter the region, the more the similarity decreases. Click here for file

Additional file 3 **Visualization of Foldback4 family with GraphDNA**. Each line corresponds to a different sequence of FoldBack4. All sequences start at the same coordinate. The sequence FB4_1 is the reference sequence. Click here for file

Additional file 4 **Visualization of Foldback4 family with Recon**. As in the results displayed by ModuleOrganizer, each module has its own texture with graphDNA. The result corresponds to the final output of Recon: all similar parts have been associated in regard to the Recon results. Click here for file

Additional file 5 **Visualization of Foldback4 family using intermediate results of Recon**. As in the results displayed by ModuleOrganizer, each module has its own texture with graphDNA. The intermediate results correspond to the enumeration of all similar parts recognized by BLAST comparisons. Click here for file
