# Supplementary figures and images for: ModuleOrganizer: detecting modules in families of transposable elements
Source: BMC Bioinformatics. 2010 Sep 22;11:474. doi: 10.1186/1471-2105-11-474 (PMC2955051; doi:10.1186/1471-2105-11-474)

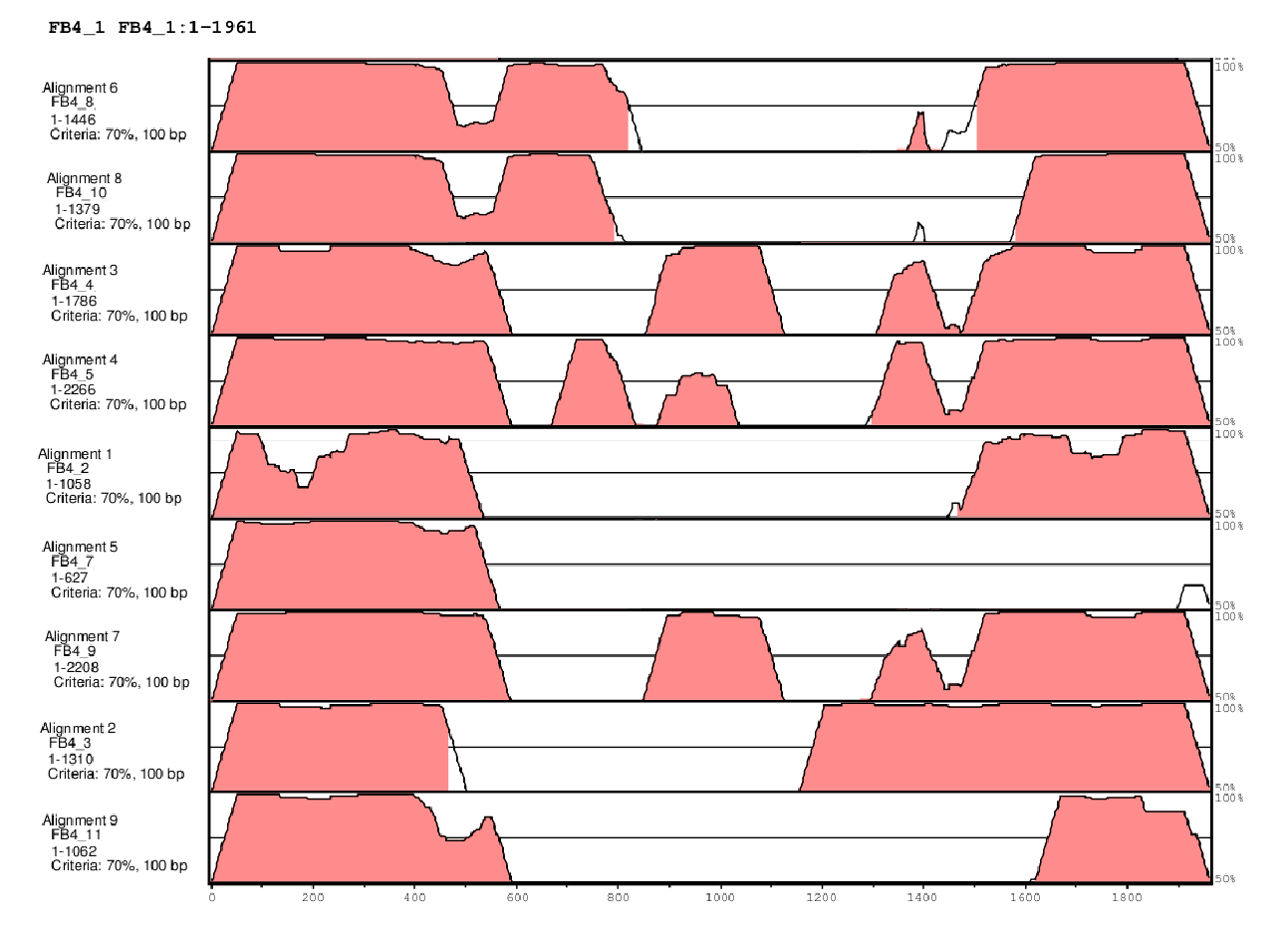

Supplement: Additional file 1 — Visualization of Foldback4 family with VISTA. We chose FB4_1 as reference sequence. The bright red zones correspond to high similarity regions and the white zones correspond to low similarity region. The alignment criteria of VISTA are fixed in the software. [file 1471-2105-11-474-S1.PNG]

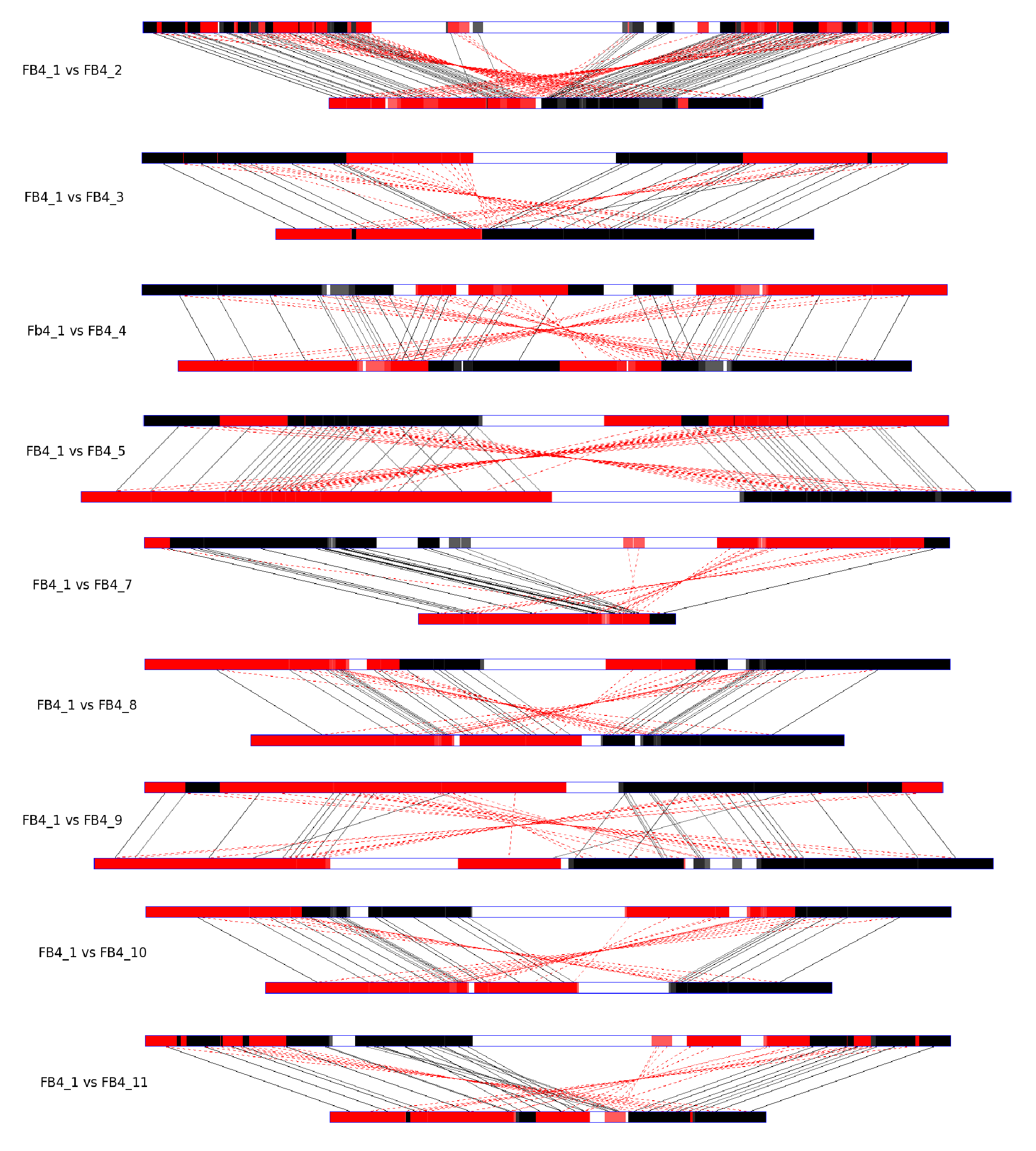

Supplement: Additional file 2 — Visualization of Foldback4 family with GATA. The reference sequence is FB4_1. The black and gray rectangles correspond to regions matched in the same direction and the red and bright red ones correspond to regions matched in reverse direction. The brighter the region, the more the similarity decreases. [file 1471-2105-11-474-S2.PNG]

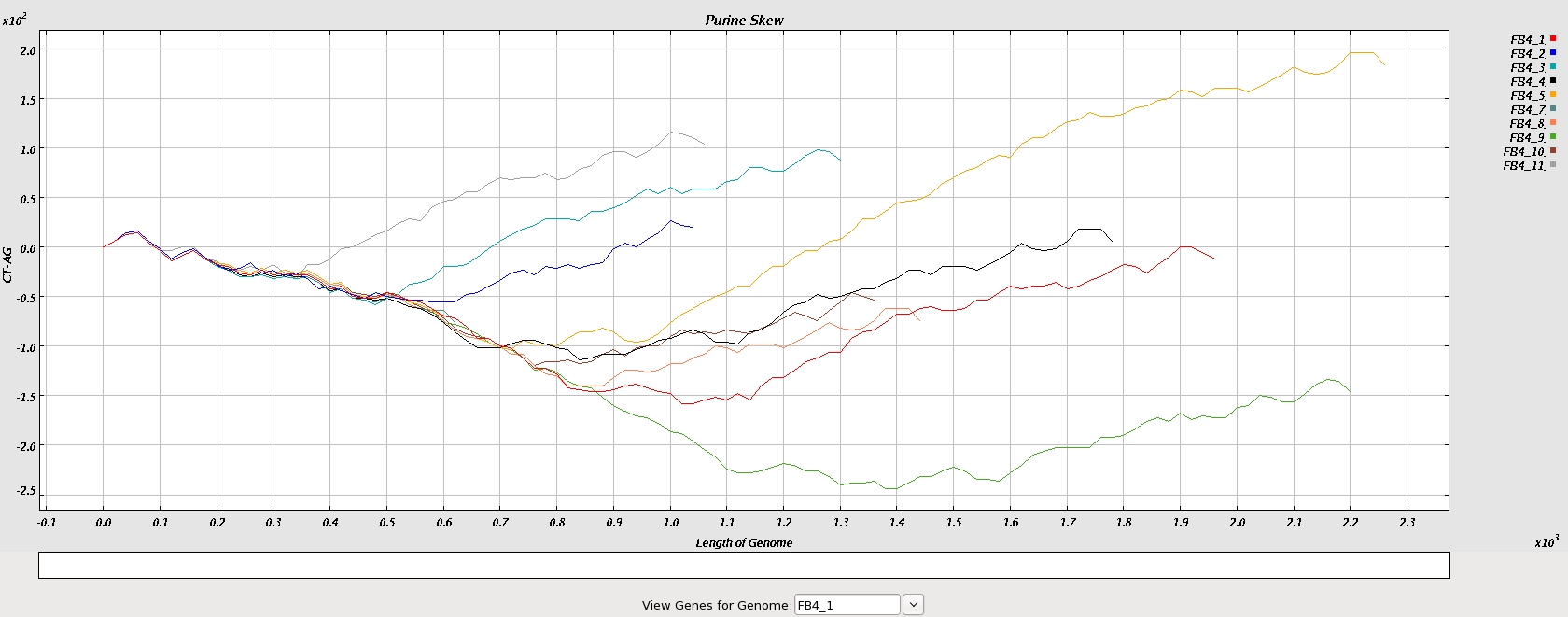

Supplement: Additional file 3 — Visualization of Foldback4 family with GraphDNA. Each line corresponds to a different sequence of FoldBack4. All sequences start at the same coordinate. The sequence FB4_1 is the reference sequence. [file 1471-2105-11-474-S3.PNG]

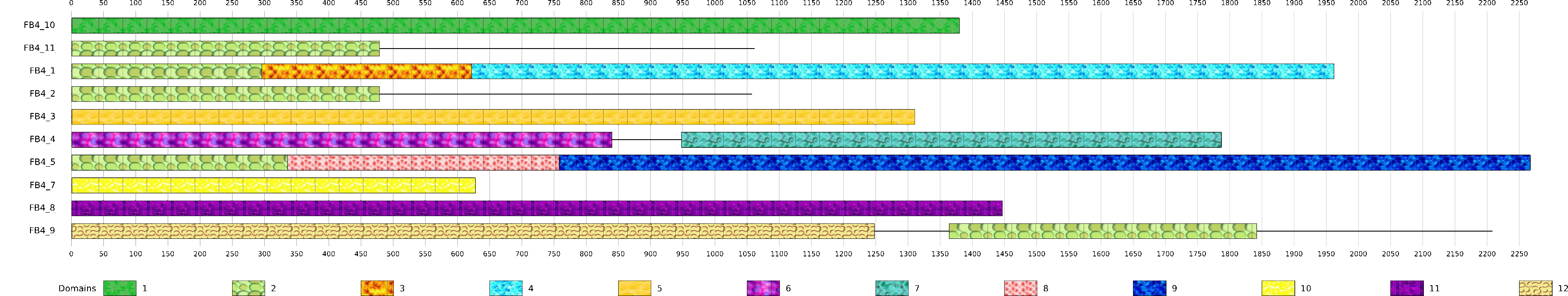

Supplement: Additional file 4 — Visualization of Foldback4 family with Recon. As in the results displayed by ModuleOrganizer, each module has its own texture with graphDNA. The result corresponds to the final output of Recon: all similar parts have been associated in regard to the Recon results. [file 1471-2105-11-474-S4.PNG]

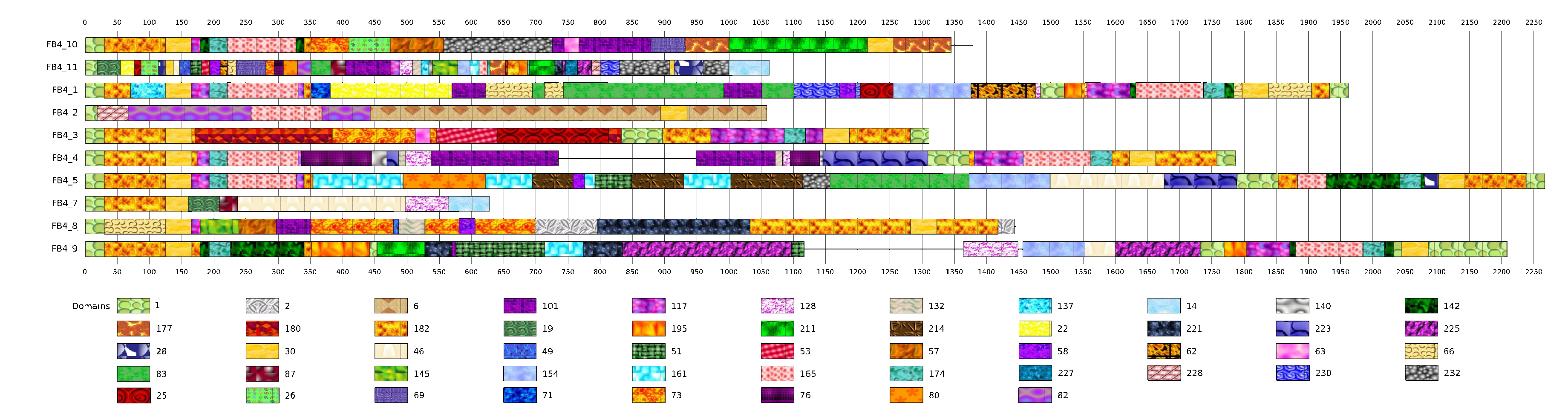

Supplement: Additional file 5 — Visualization of Foldback4 family using intermediate results of Recon. As in the results displayed by ModuleOrganizer, each module has its own texture with graphDNA. The intermediate results correspond to the enumeration of all similar parts recognized by BLAST comparisons. [file 1471-2105-11-474-S5.PNG]
